# Developing a core outcome set for gender-affirming healthcare in transgender and gender diverse adults in Sweden using the Delphi approach: a study protocol

**DOI:** 10.1136/bmjopen-2024-098300

**Published:** 2025-07-08

**Authors:** Lisann Dahlén, Klara Pettersson, Frank Berglund, Owe Bodlund, Cecilia Dhejne, Maria Elfving, Louise Frisén, Maria Halldin-Stenlid, Jenny Holmberg, Mats Holmberg, Jens Högström, Malin Indremo, Levi Karvonen, Gunnar Kratz, Ulrika Nygren, Gennaro Selvaggi, Alkistis Skalkidou, Edward Summanen, Maria Södersten, Åsa Tivesten, Kristen D Clark, Fotios C Papadopoulos

**Affiliations:** 1 Department of Medical Sciences, Clinical Psychiatry, Uppsala Universitet, Uppsala, Sweden; 2 Patient association for transgender people, Stockholm, Sweden; 3 Department of Clinical Sciences, Psychiatry, Umeå University, Umeå, Sweden; 4 Department of Psychiatry, University Hospital of Umeå, Umeå, Sweden; 5 Department of Medicine, Huddinge, Karolinska Institutet, Stockholm, Sweden; 6 ANOVA, Karolinska University Hospital, Stockholm, Sweden; 7 Department of Clinical Sciences, Pediatrics, Lund University, Lund, Sweden; 8 Department of Clinical Neuroscience, Karolinska Institute, Stockholm, Sweden; 9 Stockholm Health Care Services, Region Stockholm, Stockholm, Sweden; 10 Department of Women's and Children's Health, Karolinska Institute, Stockholm, Sweden; 11 Department of Speech-Language Pathology, University Hospital of Umeå, Umeå, Sweden; 12 Department of Clinical Sciences, Umeå University, Umeå, Sweden; 13 The Swedish Federation for Lesbian, Gay, Bisexual, Transgender, Queer and Intersex Rights (RFSL), Stockholm, Sweden; 14 Department of Biomedical and Clinical Sciences, Linköping University, Linköping, Sweden; 15 Speech and Language Pathology, Karolinska University Hospital, Stockholm, Sweden; 16 Department of Clinical Science, Intervention and Technology, Karolinska Institute, Stockholm, Sweden; 17 Department of Plastic Surgery, Institute of Clinical Sciences, Sahlgrenska Academy, University of Gothenburg, Sahlgrenska University Hospital, Gothenburg, Sweden; 18 Department of Women's and Children’s Health, Uppsala University, Uppsala, Sweden; 19 Transammans, Stockholm, Sweden; 20 Department of Endocrinology, Sahlgrenska University Hospital, Goteborg, Sweden; 21 Wallenberg Laboratory for Cardiovascular and Metabolic Research, Department of Molecular and Clinical Medicine, Institute of Medicine, Sahlgrenska Academy, University of Gothenburg, Gothenburg, Sweden

**Keywords:** Health Services for Transgender Persons, Patient Reported Outcome Measures, Protocols & guidelines

## Abstract

**Introduction:**

Despite an increasing amount of research related to gender-affirming treatment (GAT) outcomes among transgender and gender-diverse (TGD) people (ie, people who experience discomfort or distress in the misalignment between their gender and sex assigned at birth) in recent years, the evidence base for current recommendations is suboptimal. One contributing factor is the heterogeneity in the outcomes and outcome measures used. This study seeks to address this challenge by developing a foundational core outcome set (COS) to be used for TGD adults receiving GAT in Sweden.

**Methods:**

Recommendations from the Core Outcome Measures in Effectiveness Trials initiative will be used to address this aim in four phases. Phase 1, an umbrella review of peer-reviewed literature and international guidelines in GAT will be conducted to identify relevant outcomes. In phase 2, we will solicit input from TGD individuals through the review of patient and interest organisations’ reports and an anonymous survey to identify outcomes of personal significance. In phase 3, using the Delphi method, 2–3 rounds of assessment will be conducted where researchers, healthcare professionals, policy-makers and TGD adults rate the identified outcomes by perceived importance. In phase 4, a consensus meeting will convene representatives from all stakeholder groups to finalise the COS.

**Analysis:**

The results of this study will consist of a COS for GAT regarding TGD adults in Sweden. Participant survey responses will be evaluated using interpretive analysis to identify core outcomes. During each of the Delphi rounds, Likert-type scale ratings will be aggregated for outcomes to advance or be eliminated in each round.

**Ethics and dissemination:**

The study has received ethical approval by the Swedish Ethical Review Authority (Umeå medicine department, Registration number: 2024-04672-01). The results of this study will be published open-access and disseminated through TGD interest organisations and a Swedish research network for gender dysphoria.

**Trial registration number:**

COMET registration number 3223.

**Strengths and limitations of this study**

- The integration of input from both patients and clinician stakeholder groups strengthens the development of this core outcome set, thereby enhancing its relevance and utility for real-world practice.
- The use of multiple Delphi rounds, combined with feedback from a pre-Delphi round, supports the development of a well-rounded and broadly agreed on set of core outcomes.
- Although efforts are made to maintain engagement (such as personalised reminders), the reliance on voluntary participation in multiple Delphi rounds and a final consensus meeting may result in participant attrition, potentially affecting the final consensus.
- Given the broad scope of the study, narrowing down outcomes to a manageable set of core variables may inadvertently exclude potentially significant outcomes, especially those less widely recognised across the multiple clinical specialties involved.

## Introduction

 Since 2011, a substantial increase in transgender and gender-diverse (TGD) people seeking gender-affirming treatment (GAT) has been observed worldwide,^1 2^ including Sweden.^3 4^ In parallel with this increase, several knowledge gaps in GAT have been identified as priorities for future investigation.^5^,^8^

A lack of consensus on which essential outcomes should be measured in GAT research and clinical care is one particularly notable concern.^9^,^11^ There is also considerable heterogeneity in the measurement of outcomes applied in GAT research and clinical care.^9^,^11^ For example, in a systematic review of 78 studies on risk and protective factors for self-harm in TGD people, Bird *et al*^12^ found a diverse range of reported outcomes and measurement instruments, including ten different rating scales for depression. Similarly, a review on the effects of gender-affirming hormonal treatment by The Swedish Agency for Health Technology Assessment and Assessment of Social Services found that different versions of varying rating scales of gender dysphoria were used across and within studies.^13^ Additionally, psychosocial functioning is often used as an outcome for determining the effectiveness of GAT; however, there is ambiguity on which aspects of this broad construct are relevant and how they should be assessed.^6^ These gaps are interwoven. The lack of consensus on what to measure and how to measure it contributes to a fragmented understanding of psychosocial functioning as well as other outcomes in GAT for TGD individuals. This fragmentation obstructs the advancement of evidence-based treatment guidelines and limits the ability of researchers and clinicians to evaluate the effectiveness of GAT interventions.

With the recent surge in TGD people seeking care and identification of existing knowledge gaps in the field, a critical need for advancements in evidence-based treatment recommendations has become clear. As a response, National Highly Specialised Care Centres for gender dysphoria were introduced in Sweden in January 2024. This involved the integration of multiple disciplines who provide GAT to improve delivery of comprehensive care and create a stronger foundation for research. These centres include multidisciplinary expertise within psychiatry, endocrinology, voice and communication therapy, and external genital and vocal fold surgery.^14^ In the creation of the National Specialised Medical Care Centres, there emerges the opportunity and obligation for multidisciplinary collaboration and research within this field.

The need for common outcomes and measurement approaches is not new to the field of TGD health and GAT and, in fact, is a common barrier throughout healthcare delivery globally. To address this challenge, the Core Outcome Measures in Effectiveness Trials (COMET) initiative was launched in 2010 with the objective of providing guidance towards the development and application of a standardised set of outcomes through multidisciplinary collaboration and stakeholder involvement.^15^ The development of core outcome sets (COSs) involves researchers, clinicians and patients who engage in a process where they ultimately reach consensus on the most important outcomes for a specific condition to be measured and reported by all researchers in the corresponding field, thereby providing the basis for a more effective synthesis of future research findings. In accordance with the COMET guidelines, this study aims to develop a foundational COS intended for use across the broad domain of GAT. The purpose of this COS is to establish a base set of outcomes that should be measured and reported in both clinical research and in routine care involving TGD individuals receiving GAT, in contrast to other protocols, such as GenderCOS, which sought to identify a COS for genital surgeries for TGD individuals.^16^ This COS is not intended to replace more specific and nuanced COS necessary within specialties (eg, surgery, endocrinology), but rather to complement and unify them by ensuring that key outcomes, particularly those related to psychosocial functioning, mental health and overall treatment impact, are captured consistently across them. Subsequently, the scope of this COS is deliberately broad, reflecting the multidisciplinary construction of GAT and the need for a shared framework across specialties. Future COS initiatives may build on this foundation to develop domain-specific extensions tailored to particular interventions or specialties.

## Materials and methods

### Administrative information

In the development and implementation of this study, the COMET guidelines will be applied.^15^ Similarly, the information provided in this protocol is based on the ACcurate COnsensus Reporting Document.^17 18^ The present study is registered in the COMET database with the registration number 3223 under the name ‘Developing a Core Outcome Set for Gender Dysphoria in Sweden’.^19^ The study is anticipated to start in June 2024 and end in May 2025.

### Patient and public involvement

A study oversight committee comprising representatives of three different Swedish interest organisations for TGD people, as well as healthcare professionals and researchers from all academic clinical centres providing gender-affirming healthcare services in Sweden, will provide guidance for our research. Additionally, a project management group including experienced researchers, PhD students and research assistants will oversee the day-to-day operations.

There are two main groups of stakeholders that will be involved as participants:

1. Experts by experience: adults (18 years or older) in Sweden with trans experience will be invited to participate. Trans experience is defined in the present study as one who identifies as transgender, non-binary or having symptoms of gender dysphoria, either currently or in the past. Additionally, as our goal is to identify core outcomes for GAT, participants must be (a) waiting to begin, (b) currently undergoing or (c) have previously accessed GAT.
2. Experts by profession: clinical experts, researchers and policy-makers in the field of gender-affirming healthcare will be invited to participate, more precisely:
  1. Clinical experts in gender-affirming healthcare in Sweden, including psychologists, psychiatrists, surgeons, endocrinologists, speech and language pathologists, nurses and gynaecologists that have been actively involved in gender-affirming healthcare for at least 1 year.
  2. Researchers actively engaged in gender dysphoria research in Sweden.
  3. Policy-makers from government agencies who are actively involved in decision-making processes and the development of guidelines for gender-affirming healthcare.

### Recruitment

Interest organisations representing TGD people will be contacted to facilitate the recruitment of this stakeholder group. They will be provided with posters and asked to spread information about the study through their social media accounts and local group meetings. Interested individuals can follow a link to an anonymous online survey with questions about personal experiences with gender-affirming healthcare. If they are interested in participating in other parts of the study, they will have the opportunity to register their email address at the end of the survey. Email addresses will be saved separately without connection to any of the anonymous survey answers.

Healthcare professionals will be recruited from gender clinics and treatment centres across Sweden. Additionally, researchers will be recruited through a national research network focused on gender dysphoria in Sweden. Policy-makers from government agencies will be contacted at conferences and by email. Healthcare professionals, researchers and policy-makers will be provided with information about the study via email and the opportunity to register their interest in participation by completing a short online form.

The goal is to recruit a minimum of 30 participants from each stakeholder group, as this has been shown to be the minimum sample size for high replicability and is, therefore, recommended.^20^ This estimate is inclusive of the anticipated attrition that may occur through the process. However, the generalisability of the COS improves with increased participation; therefore, there will be no restrictions on the number of participants.^15^

### Development of the COS

The process of developing the COS will involve four phases: the umbrella review, the identification of outcomes relevant for TGD people, engagement on prioritisation by all stakeholder groups, and a final consensus on core outcomes related to GAT for TGD adults.

#### Phase 1: umbrella review

An umbrella review using the PubMed database will be conducted to compile a comprehensive list of outcomes assessed in clinical research (ie, studies involving participants in clinical settings) on gender dysphoria. Inclusion criteria are that studies must be: a systematic review or umbrella review, involve adult participants, published during the past 2 years (2023–2024) and published in English, Swedish or German. Therefore, reviews involving adolescents or youth (<18 years) were excluded. The specific search string will read as follows: ‘gender dysphoria’ OR ‘gender identity’ OR ‘transsexualism’ NOT ‘adolescen*’ NOT ‘child*’. Additionally, recently published Swedish reviews on GAT and any national or international guidelines for GAT will also be included in the review. Information on study characteristics, outcomes and outcome measurement instruments will be extracted.

#### Phase 2: survey

In order to capture all outcomes of personal significance to people with trans experience, published reports by TGD interest organisations will be reviewed. Additionally, direct input from TGD people will be solicited in an anonymous online survey consisting of questions about their hopes, goals and experiences with GAT. The survey will address these questions in context to assessment, hormonal treatment, surgical interventions, voice and communication training and a category called ‘other treatments’ where participants can describe their experiences with treatments not included in the prior categories. The survey will be available in both English and Swedish to promote inclusivity and broaden participation, especially among non-native Swedish speakers, such as migrants living in Sweden (online supplemental file 1). Informed consent is waived for this part of the study, per the Swedish Ethical Review Authority (Umeå medicine department, Registration number: 2024-04672-01). Participants will be able to enter their email address if they are interested in participating in phase 3 of this study. Depending on the number and depth of responses to the survey, a decision will be made about the need for focus groups.

##### Survey analysis

Interpretive analysis is described as the most suitable approach for identifying treatment outcomes from participants’ direct wording^21^; therefore, this analytical approach will be applied to identify outcomes based on the participants’ responses to the open-response survey questions. The open-text responses will be analysed inductively line-by-line. The identified outcomes from this phase will be integrated and/or synthesised into the outcomes identified during the umbrella review (phase 1). Direct quotes from participant responses will be extracted as examples.

##### Condensing the list of outcomes

The list of outcomes identified in phases 1 and 2 will be reviewed to remove duplicates and merge similar outcomes. Three study personnel will review all outcomes for proposed synthesis, with a fourth individual who will resolve any disagreements. Subsequently, the outcomes will be categorised according to the COMET taxonomy which was specifically developed for this purpose.^22^ In the next step, a plain language description will be provided for each outcome, with the assistance of the previously extracted quotations from the survey of TGD participants.

##### Pre-Delphi

The number of outcomes will be reduced through a pre-Delphi round within the oversight committee. This will be conducted to avoid attrition of participants due to lengthy Delphi surveys.^23^ All outcomes from the previously condensed list will be rated on a Likert-type scale from 1 (of no importance’) to 5 (‘very important’) based on their perceived importance by the members of the oversight committee. The 25 highest rated outcomes by representatives of TGD interest organisations and the 25 highest rated outcomes by the other members of the oversight committee, that is, clinicians and researchers, will be combined into the final list of outcomes to be included in the next Delphi process. This list will finally be discussed and agreed on in a meeting with the oversight committee to ensure that no important outcomes are being overlooked. Informed consent for this and the subsequent Delphi rounds will be obtained at the beginning of the survey, where participants can indicate whether they ‘yes’—consent to participation or ‘no’—do not consent. If participants indicate ‘no’, the survey will end.

### Phase 3: Delphi rounds

The Delphi method is commonly used in COS development to reach consensus among participants.^15^ The method consists of multiple iterations of item rating and subsequent rating adjustment based on feedback from other participants.^24^

In this study, two to three Delphi rounds will be conducted online using the RedCap platform. In each round, participants of the two stakeholder groups will anonymously rate each outcome based on its presumed importance on a 5-point Likert scale,^25^ with 1 indicating that the outcome is considered ‘of no importance’, 2 ‘of little importance’, 3 ‘neutral’, 4 ‘important’ and 5 ‘very important’. An ‘unable to score’ option will also be available in case a participant does not feel comfortable scoring a certain outcome due to inexperience or other personal reasons. After rating each outcome, each participant will have the option to justify their rating with an open-text response. The outcomes will be organised within the following categories: general outcomes (which should be measured in all GAT research independent of the discipline), endocrinology, voice and communication, and surgery.

Each round will remain open for 3 weeks. At the beginning of each round, participants will receive instructions on how to complete the survey and a reminder of the overall aim to achieve consensus on a core set of outcomes.

#### Round 1

Participants will initially be asked to suggest a maximum number of five outcomes that they consider most important in gender-affirming healthcare. This approach has proven to be important in ensuring that no critical outcomes are missed.^26^ Any additional item named at least twice will be added to the subsequent Delphi round. Participants will then rate the importance of each predefined outcome on a 5-point Likert scale^25^ and have the opportunity to justify their rating, as described above.

A personalised reminder will be sent to the participants weekly after opening the first survey round. If less than 80% of participants respond within the initial 3-week period that this round is open, the research team may consider extending the response period by an additional week.

#### Round 2

Participants will rerate all outcomes, this time after being provided with feedback from the results of the previous round from all stakeholder groups, presented as histograms that display each group’s collective voting and justifications from open-text responses, as this approach has been found to improve stakeholders’ understanding of the Delphi round results.^27^ Participants will be asked if they are willing to participate in a consensus meeting to finalise the list of core outcomes.

Based on the ratings in round 2, the list will be shortened according to the following criteria: All outcomes that 75% of respondents in each group are scoring 4 or higher and <15% of respondents in each group are scoring 2 or lower will be included in the consensus meeting.

A third Delphi round will be conducted if the results after round 2 show significant variability in ratings or the consensus between stakeholder groups remains low.

### Phase 4: consensus meeting

The final step in the consensus process will be a meeting with representatives from all stakeholder groups to finalise the COS. Prior to the meeting, the results of the last Delphi round will be sent to all participants including the final ratings of all the remaining outcomes. A preparatory meeting will be held with the TGD participants to empower them to voice their views during the consensus meeting.

The meeting will be led by an experienced facilitator and begin with a reminder of the scope of the COS and a summary of the work completed to date. The nominal group technique is commonly used as the final step in achieving consensus on the COS.^15^ This technique allows for the initial consideration of all opinions before a final voting process is conducted.^28^ Following this approach, all participants will be given the opportunity to express their views on the remaining outcomes. Subsequently, a final voting process will be conducted during which all participants will rank the remaining outcomes according to their perceived importance on a 5-point Likert scale (similar to the ratings in the Delphi rounds). Any outcome rated 4 or higher by at least 80% of participants will be included in the final COS.

The goal is to identify a minimum of four and a maximum of six outcomes in the general category (outcomes that should be measured in all GAT research independent of the discipline) and 2–4 outcomes for each of the other categories, that is, endocrinology, voice and communication, and surgery, at the end of the consensus process. There is no definitive recommendation regarding the ideal number of outcomes in a COS, and no clear relationship between the number of outcomes and their adoption could be found.^29^ Accordingly, the decision regarding the minimum and maximum number of outcomes is based on balancing the potential burden on participants to complete outcome measurements with the risk of overlooking important outcomes. In the event that more than six outcomes per category receive a rating of 4 or higher from at least 80% of participants, it may be necessary to adjust the threshold to a higher percentage.

### Ethics and dissemination

The study has received ethical approval by the Swedish Ethical Review Authority (Umeå medicine department, Registration number: 2024-04672-01). Based on a recommendation from TGD representatives in the oversight committee and the, the anonymous survey for TGD participants in phase 2 of this study will not require participants to complete an informed consent form. This decision was taken to ensure complete participant anonymity. Informed consent will be obtained from all other individual participants included in the study.

All procedures performed in studies involving human participants will be in accordance with the ethical standards of the institutional and/or national research committee and with the 1964 Declaration of Helsinki and its later amendments or comparable ethical standards.

The results of this study will be published open-access and disseminated through TGD interest organisations and a Swedish research network for gender dysphoria.

## Supplementary material

10.1136/bmjopen-2024-098300 online supplemental file 1
