# Supplementary material for: Developing a core outcome set for gender-affirming healthcare in transgender and gender diverse adults in Sweden using the Delphi approach: a study protocol
Source: BMJ Open. 2025 Jul 8;15(7):e098300. doi: 10.1136/bmjopen-2024-098300 (PMC12243596; doi:10.1136/bmjopen-2024-098300)
Supplement: online supplemental file 1 [file bmjopen-15-7-s001.pdf]

Core Outcome Set för könsdysfori

If you want to switch to English, click the button on the right hand side.

Hej!

Tack för att du är intresserad av vår studie om att ta fram ett Core Outcome Set för könsdysfori!

Ett Core Outcome Set är en lista med viktiga utfall (resultat av en insats) som syftar till att göra forskningsresultat inom könsdysfori mer jämförbara och därmed kunna ge bättre vård till patienter inom den könsbekräftande vården i Sverige.

Den här enkäten vänder sig till personer med transerfarenhet som antingen väntar på, har en aktuell eller tidigare kontakt med den könsbekräftande vården. Om du har påbörjat och valt att avsluta en utredning eller behandling för könsdysfori eller om du har erfarenheter som du själv beskriver som detransition, retransition eller ånger, är du också välkommen att svara på enkäten.

Lite bakgrundsinformation om Core Outcome Sets:

För att utveckla vården och göra den så bra som möjligt behöver vi veta vad som fungerar bra respektive mindre bra. Olika studier mäter ofta olika saker, vilket gör det svårt att jämföra resultaten. Här kommer Core Outcome Sets in i bilden.

Ett Core Outcome Set är en lista med viktiga resultat som alla forskare ska mäta och rapportera i sina studier inom ett visst område. Detta gör det lättare att jämföra och kombinera forskningsresultat, vilket kommer att förbättra informationen om vilka behandlingar som fungerar bra respektive mindre bra och därmed hjälpa patienter att fatta bättre informerade beslut om sin vård.

Vi använder enkäter och involverar experter, inklusive patienter, vårdgivare och forskare, för att ta fram ett Core Outcome Set. Din medverkan är viktig för att säkerställa att dessa resultat är relevanta för personer med könsdysfori!

Vi uppskattar din hjälp! Om du vill veta mer eller har frågor, kontakta oss via lisann.dahlen@uu.se.

Det är frivilligt att delta och dina svar på den här enkäten är anonyma.

Om du vill veta mer om Core Outcome Sets, finns mer information här och en kort video (3:22min) på engelska här. Mer information på svenska finns på SBU:s webbsida.

Genom hela denna undersökning kommer du att se termen "transperson". Vi använder denna term på ett brett sätt för att beskriva alla individer som identifierar sig som transpersoner, ickebinära, agender eller någon annan könsidentitet som skiljer sig från det kön de tilldelades vid födseln.

Enkäten genomförs av Uppsala universitet inom ramen för forskning om könsdysfori.

Inledning

Är du 18 år eller äldre?

○ Ja
○ Nej

Beskriver du dig själv som transperson eller har du symptom på könsdysfori eller könsinkongruens?

○ Ja
○ Nej

Har du tidigare beskrivit dig själv som transperson, men beskriver dig inte längre så? Eller har dina tidigare symptom på könsdysfori/könsinkongruens försvunnit/mildrats?

○ Ja
○ Nej

Hur gammal är du?	◯ 18- 25 ◯ 26- 35 ◯ 36- 50<br>◯ 51- 65 ◯ 65+
Vad tycker du bäst beskriver din nuvarande könsidentitet?	☐ Agender<br>☐ Genderqueer/genderfluid<br>☐ Ickebinär<br>☐ Kvinna<br>☐ Man<br>☐ Transkvinna<br>☐ Transman<br>☐ Osäker<br>☐ Annat
	________________________
Vilket kön tilldelades du vid födseln (alltså vilket kön registrerades för dig juridiskt, i journal, folkbokföring etc.)?	◯ Kvinna<br>◯ Man
Har du en medfödd intersexvariation (ibland även kallat intersextillstånd/DSD)?	◯ Ja<br>◯ Nej
Beskriv tillståndet och/eller vilken diagnos du har fått	________________________
Har du haft minst ett möte med en könsdysforimottagning (i Sverige)?	◯ Ja<br>◯ Nej, men jag står på väntelistan<br>◯ Nej, jag står inte på väntelistan
Vilket år ställdes du på väntelista (t.ex. 2022)?	________________________
Kan du beskriva skälet till att du inte står på väntelistan?	________________________
Har du genomgått en utredning för könsdysfori på en svensk könsdysforimottagning?	◯ Ja<br>◯ Jag är i processen<br>◯ Nej, jag planerar att starta processen i framtiden<br>◯ Nej, jag gjorde en utredning på en könsdysforimottagning utomlands<br>◯ Nej, jag önskar ingen utredning för könsdysfori
Jag påbörjade min könsdysforiutredning	◯ före 18 års ålder<br>◯ vid eller efter 18 års ålder
Ställde teamet en könsdysforidiagnos?<br><br>Dessa diagnoskoder används i vården idag utifrån ICD10, i den uppdaterade versionen ICD11 kommer ett annat språkbruk användas. På många könsdysforimottagningar anpassar man ordvalet redan idag och skriver efter F648 något som stämmer för personen det gäller tex. "ickebinär".	◯ F640 - Transsexualism<br>◯ F648 - andra specificerade könsidentitetsstörningar<br>◯ Annan diagnos<br>◯ Ingen diagnos<br>◯ Jag vet inte
Vilken?	________________________

Kan du beskriva varför du inte fick en könsdysforidiagnos? Håller du med om detta beslut?

____

Har du någonsin fått någon könsbekräftande behandling?

Detta kan inkludera hormoner, kirurgi, röst/kommunikationsträning (träffat logoped), bekräftande hjälpmedel (penisprotes, bröstproteser eller andra icke-kirurgiska hjälpmedel), psykologiskt eller socialt stöd för att lindra eller hantera könsdysfori.

○ Ja
○ Nej

Kan du beskriva varför du inte har fått behandlingar?

Detta kan bero på väntetider, bedömningen från könsdysforimottagningen, ekonomiska bekymmer eller någon annan anledning.

____

Önskar du några behandlingar i framtiden?

Detta kan inkludera hormoner, kirurgi, röst/kommunikationsträning (att träffa logoped) eller psykologiskt stöd.

Om du redan genomgått vissa behandlingar, svara ja om du vill ha andra behandlingar än de du redan får/har fått.

○ Ja
○ Nej
○ Osäker

Kan du beskriva varför du inte önskar några behandlingar?

Detta kan bero på att du inte känner att du behöver några behandlingar, ditt allmänna hälsotillstånd eller någon annan anledning.

____

Har du fått någon könsbekräftande behandling utanför de svenska könsdysforimottagningarna?

○ Ja
○ Nej

Behandling utanför de svenska könsdysforimottagningarna

Har du fått, eller övervägt att söka könsbekräftande behandling i ett annat lands offentliga sjukvårdssystem?

○ Ja
○ Nej

Vilka interventioner har du fått och varifrån?

____

Vilka interventioner har du övervägt men inte fått?

____

Har du fått, eller övervägt att söka, könsbekräftande behandling från privata vårdmottagningar, såsom GenderGP eller en annan privat mottagning?

○ Ja
○ Nej

Vilka interventioner har du fått och varifrån?

____

Vilka interventioner har du övervägt men inte fått?

____

Har du använt, eller övervägt att använda, egenmedicinering (DIY) eller andra metoder som inte övervakas av vårdpersonal?

Detta kan inkludera kroppsförändringar som du gjort själv, hormoner eller silikon köpt online, eller andra könsbekräftande interventioner utan recept/remiss/eller övervakning av vårdpersonal.

○ Ja
○ Nej

Vilka interventioner har du fått och varifrån?

____

Vilka interventioner har du övervägt men inte fått?

____

Har du övervägt någon av följande källor till könsbekräftande behandling utanför de svenska könsdysforimottagningarna?

☐ Från en privat mottagning
☐ Självmedicinering (DIY) eller andra metoder som inte övervakas av vårdpersonal
☐ En annan källa
☐ Jag har inte övervägt att söka vård från andra källor än de svenska könsdysforimottagningarna.

Vilka interventioner har du övervägt? Vad har hindrat dig från att få dessa behandlingar?

____

Utredning

Hur länge har du haft din könsdysforidiagnos? Om du inte fick en diagnos, hur länge sedan var det du avslutade din könsdysforiutredning?

○ Mindre än 1 år
○ 2-4 år
○ 5+ år

Vilka förändringar önskade du att din könsdysforiutredning skulle leda till?

Exempelvis emotionella förändringar (känslor/mående), sociala förändringar (hur ditt liv ser ut eller kontakt med andra/din omgivning) eller fysiska förändringar (utseende/kropp).

____

Har könsdysforiutredningen motsvarat dina förväntningar? Varför ja eller nej?

____

Upplevde du några oväntade eller oönskade förändringar efter din könsdysforiutredning?

Exempelvis emotionella förändringar (känslor/mående), sociala förändringar (hur ditt liv ser ut eller kontakt med andra/din omgivning) eller fysiska förändringar (utseende/kropp).

____

Hur skulle du sammanfatta din upplevelse av kontakten med könsdysforimottagningen under utredningen?

____

Vilka förväntningar har du inför din utredning?

____

Vilka förändringar hoppas du på som ett resultat av din utredning?

____

Finns det förändringar kopplade till din utredning som du inte vill ha eller är orolig för?

____

Hormonbehandling

Har du fått könsbekräftande hormonbehandling (HRT)?

- ◯ Ja
- ◯ Nej, men jag står på väntelistan för att börja hormonbehandling (HRT).
- ◯ Nej, men jag önskar få hormonbehandling (HRT) i framtiden (inte på väntelistan).
- ◯ Nej, jag vill inte ha hormonbehandling (HRT).
- ◯ Nej, jag är osäker om jag vill ha hormonbehandling (HRT).

Vilket år ställdes du på väntelista (t.ex. 2022)?

____

Hur länge har du haft hormonbehandling (HRT)?

- ◯ Mindre än 1 år
- ◯ 2-4 år
- ◯ 5+ år

Vilka förändringar önskade du dig som ett resultat av din hormonbehandling (HRT)?

Exempelvis emotionella förändringar (känslor/mående), sociala förändringar (hur ditt liv ser ut eller kontakt med andra/din omgivning) eller fysiska förändringar (utseende/kropp).

____

Har hormonbehandlingen (HRT) motsvarat dina förväntningar? Varför ja eller nej?

____

Upplevde du några oväntade eller oönskade förändringar efter din hormonbehandling (HRT)? Vilka?

Exempelvis emotionella förändringar (känslor/mående), sociala förändringar (hur ditt liv ser ut eller kontakt med andra/din omgivning) eller fysiska förändringar (utseende/kropp).

____

Hur skulle du sammanfatta din upplevelse av kontakten med endokrinologiska mottagningen/gynekologiska mottagningen under din hormonbehandling (HRT)?

____

Vilka förväntningar har du inför att påbörja hormonbehandling (HRT)?

____

Vilka förändringar hoppas du på som ett resultat av en hormonbehandling (HRT)?

Exempelvis emotionella förändringar (känslor/mående), sociala förändringar (hur ditt liv ser ut eller kontakt med andra/din omgivning) eller fysiska förändringar (utseende/kropp).

____

Finns det förändringar kopplade till hormonbehandling (HRT) som du inte vill ha eller är orolig för?

Exempelvis emotionella förändringar (känslor/mående), sociala förändringar (hur ditt liv ser ut eller kontakt med andra/din omgivning) eller fysiska förändringar (utseende/kropp).

____

Hur skulle du beskriva din upplevelse av eventuell kontakt med endokrinologiska mottagningen/gynekologiska mottagningen inför att påbörja din hormonbehandling (HRT)?

____

Bröstkorgskirurgi

Har du genomgått könsbekräftande bröstkorgskirurgi?

Även kallat överkroppskirurgi. Bröstkorgskirurgi inkluderar både bröstförstoring och mastektomi.

- ◯ Ja
- ◯ Nej, men jag står på väntelistan för bröstkorgskirurgi
- ◯ Nej, men jag önskar få bröstkorgskirurgi i framtiden (inte på väntelistan)
- ◯ Nej, jag vill inte ha bröstkorgskirurgi
- ◯ Nej, jag är osäker om jag vill ha bröstkorgskirurgi

Vilket år ställdes du på väntelista (t.ex. 2022)?

____

Hur länge sedan genomfördes din bröstkorgskirurgi?

- ◯ För mindre än 1 år sedan
- ◯ För 2-4 år sedan
- ◯ För 5+ år sedan

Vilka förändringar önskade du att din bröstkorgskirurgi skulle leda till?<br><br>Exempelvis emotionella förändringar (känslor/mående), sociala förändringar (hur ditt liv ser ut eller kontakt med andra/din omgivning) eller fysiska förändringar (utseende/kropp).	__________________________________
Har din upplevelse av bröstkorgskirurgi motsvarat dina förväntningar? Varför ja eller nej?	__________________________________
Upplevde du några oväntade eller oönskade förändringar efter din bröstkorgskirurgi? Vilka?<br><br>Exempelvis emotionella förändringar (känslor/mående), sociala förändringar (hur ditt liv ser ut eller kontakt med andra/din omgivning) eller fysiska förändringar (utseende/kropp).	__________________________________
Hur skulle du sammanfatta din upplevelse av kontakten med plastikkirurgiska mottagningen i samband med din bröstkorgskirurgi?	__________________________________
Vilka förväntningar har du inför bröstkorgskirurgin?	__________________________________
Vilka förändringar hoppas du på som ett resultat av bröstkorgskirurgi?<br><br>Exempelvis emotionella förändringar (känslor/mående), sociala förändringar (hur ditt liv ser ut eller kontakt med andra/din omgivning) eller fysiska förändringar (utseende/kropp).	__________________________________
Finns det förändringar kopplade till bröstkorgskirurgi som du inte vill ha eller är orolig för?<br><br>Exempelvis emotionella förändringar (känslor/mående), sociala förändringar (hur ditt liv ser ut eller kontakt med andra/din omgivning) eller fysiska förändringar (utseende/kropp).	__________________________________
Hur skulle du beskriva din upplevelse av eventuell kontakt med plastikkirurgiska mottagningen för att få bröstkorgskirurgi?	__________________________________
Underlivskirurgi	
Har du genomgått könsbekräftande underlivskirurgi?	◯ Ja<br>◯ Nej, men jag står på väntelistan för underlivskirurgi.<br>◯ Nej, men jag önskar att få underlivskirurgi i framtiden (inte på väntelistan).<br>◯ Nej, jag vill inte ha underlivskirurgi.<br>◯ Nej, jag är osäker om jag vill ha underlivskirurgi.

Vilket år ställdes du på väntelista (t.ex. 2022)? __________________________________

Hur länge sedan genomfördes din underlivskirurgi?
- ◯ För mindre än 1 år sedan
- ◯ För 2-4 år sedan
- ◯ För 5+ år sedan

Vilka förändringar önskade du att din underlivskirurgi skulle leda till?

Exempelvis emotionella förändringar (känslor/mående), sociala förändringar (hur ditt liv ser ut eller kontakt med andra/din omgivning) eller fysiska förändringar (utseende/kropp).

Har din upplevelse av underlivskirurgi motsvarat dina förväntningar? Varför ja eller nej?

Upplevde du några oväntade eller oönskade förändringar efter din underlivskirurgi?

Exempelvis emotionella förändringar (känslor/mående), sociala förändringar (hur ditt liv ser ut eller kontakt med andra/din omgivning) eller fysiska förändringar (utseende/kropp).

Hur skulle du sammanfatta din upplevelse av kontakten med plastikkirurgiska mottagningen i samband med din underlivskirurgi?

Vilka förväntningar har du inför underlivskirurgin?

Vilka förändringar hoppas du på som ett resultat av din underlivskirurgi?

Exempelvis emotionella förändringar (känslor/mående), sociala förändringar (hur ditt liv ser ut eller kontakt med andra/din omgivning) eller fysiska förändringar (utseende/kropp).

Finns det förändringar kopplade till underlivskirurgi som du inte vill ha eller är orolig för?

Exempelvis emotionella förändringar (känslor/mående), sociala förändringar (hur ditt liv ser ut eller kontakt med andra/din omgivning) eller fysiska förändringar (utseende/kropp).

Hur skulle du beskriva din upplevelse av eventuell kontakt med plastikkirurgiska mottagningen inför att påbörja underlivskirurgi?

Röst- eller kommunikationsträning

Har du någonsin fått könsbekräftande röst- eller kommunikationsträning (träffat logoped)?	◯ Ja<br>◯ Nej, men jag står på väntelistan för röst-/kommunikationsträning<br>◯ Nej, men jag önskar att få röst-/kommunikationsträning i framtiden (inte på väntelistan)<br>◯ Nej, jag vill inte ha röst-/kommunikationsträning<br>◯ Nej, jag är osäker om jag vill ha röst-/kommunikationsträning
Vilket år ställdes du på väntelista (t.ex. 2022)?	__________________________
Hur länge sedan hade du röst-/kommunikationsträning?	◯ För mindre än 1 år sedan<br>◯ För 2-4 år sedan<br>◯ För 5+ år sedan
Vilka förändringar önskade du att din röst-/kommunikationsträning skulle leda till?<br><br>Exempelvis emotionella förändringar (känslor/mående), sociala förändringar (hur ditt liv ser ut eller kontakt med andra/din omgivning) eller fysiska förändringar (utseende/kropp).	__________________________________
Har din upplevelse av röst-/kommunikationsträning motsvarat dina förväntningar? Varför ja eller nej?	__________________________________
Upplevde du några oväntade eller oönskade förändringar efter din röst-/kommunikationsträning? Vilka?<br><br>Exempelvis emotionella förändringar (känslor/mående), sociala förändringar (hur ditt liv ser ut eller kontakt med andra/din omgivning) eller fysiska förändringar (utseende/kropp).	__________________________________
Hur skulle du sammanfatta din upplevelse av kontakten med logopedin i samband med röst-/kommunikationsträningen?	__________________________________
Vilka förväntningar har du inför röst-/kommunikationsträningen?	__________________________________
Vilka förändringar hoppas du på som ett resultat av röst-/kommunikationsträning?<br><br>Exempelvis emotionella förändringar (känslor/mående), sociala förändringar (hur ditt liv ser ut eller kontakt med andra/din omgivning) eller fysiska förändringar (utseende/kropp).	__________________________________

Finns det förändringar kopplade till röst-/kommunikationsträning som du inte vill ha eller är orolig för?

Exempelvis emotionella förändringar (känslor/mående), sociala förändringar (hur ditt liv ser ut eller kontakt med andra/din omgivning) eller fysiska förändringar (utseende/kropp).

____

Hur skulle du beskriva din upplevelse av eventuell kontakt med logopederna inför att påbörja röst-/kommunikationsträning?

____

Annan behandling eller insats

Har du någonsin fått någon annan typ av könsbekräftande behandling eller insats?

Till exempel, en hysterektomi, fertilitetsbevarande åtgärder, pubertetsblockerande behandling, ansiktsfeminiserande kirurgi, könsbekräftande hjälpmedel (t.ex. binders, penisprotes, bröstproteser) eller psykologiskt eller socialt stöd för att lindra eller hantera könsdysfori.

- ◯ Ja
- ◯ Nej, men jag står på väntelistan för andra könsbekräftande behandlingar
- ◯ Nej, men jag önskar att få andra könsbekräftande behandlingar i framtiden (inte på väntelistan)
- ◯ Nej, jag vill inte ha några andra könsbekräftande behandlingar
- ◯ Nej, jag är osäker om jag vill ha andra könsbekräftande behandlingar

Vilka behandlingar och under vilket år fick du behandlingen (t.ex. 2022)?

____

För vilka behandling(ar) och vilket år ställdes du på väntelista (t.ex. 2022)?

____

Vilka förändringar önskade du att dessa andra könsbekräftande behandlingar skulle leda till?

Exempelvis emotionella förändringar (känslor/mående), sociala förändringar (hur ditt liv ser ut eller kontakt med andra/din omgivning) eller fysiska förändringar (utseende/kropp).

____

Har din upplevelse av dessa andra könsbekräftande behandlingar motsvarat dina förväntningar? Varför ja eller nej?

____

Upplevde du några oväntade eller oönskade förändringar efter dessa andra könsbekräftande behandlingar?

Exempelvis emotionella förändringar (känslor/mående), sociala förändringar (hur ditt liv ser ut eller kontakt med andra/din omgivning) eller fysiska förändringar (utseende/kropp).

____

Hur skulle du sammanfatta din upplevelse av kontakten med vården i samband med dessa andra könsbekräftande behandlingar?

____

Vilka förväntningar har du inför dessa andra könsbekräftande behandlingar?

____

Vilka förändringar hoppas du på som ett resultat av dessa andra könsbekräftande behandlingar?

Exempelvis emotionella förändringar (känslor/mående), sociala förändringar (hur ditt liv ser ut eller kontakt med andra/din omgivning) eller fysiska förändringar (utseende/kropp).

____

Finns det förändringar kopplade till dessa andra könsbekräftande behandlingar som du inte vill ha eller är orolig för?

Exempelvis emotionella förändringar (känslor/mående), sociala förändringar (hur ditt liv ser ut eller kontakt med andra/din omgivning) eller fysiska förändringar (utseende/kropp).

____

Hur skulle du beskriva din upplevelse av eventuell kontakt med könsdysforimottagningen och de andra vårdprofessionerna för att få tillgång till dessa könsbekräftande behandlingar?

____

Behandling på en privat mottagning

Du svarade tidigare i enkäten att du hade sökt könsbekräftande behandling på en privat mottagning. Vad fick dig att söka behandling genom en privat mottagning?

____

Har det medfört några personliga svårigheter att söka könsbekräftande behandling på en privat mottagning?

____

Har det medfört några svårigheter när/om du återupptog vård inom det svenska sjukvårdssystemet efter att ha sökt könsbekräftande behandling på en privat mottagning?

Detta kan vara kopplat till könsdysforimottagningen eller andra delar av sjukvården, till exempel att söka vård för eventuella komplikationer relaterade till behandlingen som du fick på den privata mottagningen.

____

Hur skulle du beskriva kvaliteten på den vård du fått på den privata mottagningen?

- ◯ Utmärkt
- ◯ Mycket bra
- ◯ Bra
- ◯ Hyfsad
- ◯ Dålig

Egenmedicinering

Du svarade tidigare i enkäten att du hade påbörjat egenmedicinering eller annan behandling som inte övervakas av vårdpersonal. Vad fick dig att söka könsbekräftande behandling genom detta sätt?

____

Har det medfört några personliga svårigheter att söka könsbekräftande behandling genom egenmedicinering (DIY) eller andra metoder som inte övervakas av vårdpersonal?

____

Har det medfört några svårigheter när/om du återupptog vård inom det svenska sjukvårdssystemet efter att ha sökt könsbekräftande behandling som inte övervakas av vårdpersonal?

Detta kan vara kopplad till könsdysforimottagningen eller andra delar av sjukvården, till exempel att söka vård för eventuella komplikationer relaterade till behandlingen som du fick på den privata mottagningen.

____

Hur skulle du beskriva kvaliteten på den vård du fått genom metoder som inte övervakas av vårdpersonal?

- ◯ Utmärkt
- ◯ Mycket bra
- ◯ Bra
- ◯ Hyfsad
- ◯ Dålig

Frågor till dig som inte längre identifierar dig som transperson

När du identifierade dig som transperson eller när du hade könsdysforisymptom, vilken könsidentitet använde du för att beskriva dig själv?

- ☐ Agender
- ☐ Genderqueer/genderfluid
- ☐ Ickebinär
- ☐ Kvinna
- ☐ Man
- ☐ Transkvinna
- ☐ Transman
- ☐ Osäker
- ☐ Annat

____

Hur har du gjort för att bekräfta ditt kön sedan du inte längre identifierar dig som transperson eller inte längre har några könsdysforisymptom?

- ☐ Sociala förändringar (t.ex. namnbyte, pronomenbyte, kläder, frisyr)
- ☐ Avbrutit HRT
- ☐ Reversering av bröstkorgskirurgi
- ☐ Reversering av annan könsbekräftande behandling
- ☐ Fått en annan behandling (hormonell, kirurgisk eller annan)
- ☐ Jag har inte gjort något för att bekräfta mitt nuvarande kön

Reversering av vilken/vilka behandling(ar)?

____

Vilken annan/vilka andra behandling(ar)?

____

Vilka behandlingar eller andra strategier planerar du att använda i framtiden för att bekräfta ditt nuvarande kön?	☐ Sociala förändringar (t.ex. namnbyte, pronomenbyte, kläder, frisyr)<br>☐ Avbryta HRT<br>☐ Reversering av bröstkorgskirurgi<br>☐ Annan reversering av könsbekräftande behandling<br>☐ En annan behandling (hormonell, kirurgisk eller annan)<br>☐ Jag planerar inte att göra något för att bekräfta mitt nuvarande kön<br>☐ Osäker
Reversering av vilken/vilka behandling(ar)?	__________________________________
Vilken annan/vilka andra behandling(ar)?	__________________________________
Finns det åtgärder som kan vidtas för att förbättra vården du fick på könsdysforimottagningen?<br><br>Detta kan vara under utredningen eller andra delar av behandlingsprocessen.	____
Hur skulle du beskriva kvaliteten på den sjukvård du fått från könsdysforimottagningen sedan du inte längre identifierar dig som transperson eller inte längre har symtom på könsdysfori?	◯ Utmärkt<br>◯ Mycket bra<br>◯ Bra<br>◯ Hyfsad<br>◯ Dålig
Finns det åtgärder som kan vidtas för att förbättra vården du fick efter att du inte längre identifierade dig som transperson?	____
Avslutning	
Vad tycker du att patienter, vårdgivare och forskare behöver veta mer om när det gäller effekten av könsbekräftande vård?<br>Vad är viktigast att samla information om?	____
Ytterligare synpunkter	____
Hur fick du information om enkäten?	☐ Sociala medier<br>☐ Könsdysforimottagning<br>☐ Samtalsgrupp om könsidentitet eller könsdysfori<br>☐ En vän eller annan närstående<br>☐ Organisation för transpersoner<br>☐ Annat
	__________________________________

Tack så mycket för dina svar! Den här enkäten är bara första steget i vår studie, och vi vill inkludera personer med transerfarenhet under hela studieprocessen så att den slutgiltiga listan av viktiga resultat verkligen har betydelse för personer som söker vård för könsdysfori. I nästa steg ska forskare, klinisk personal och personer med transerfarenhet i 2 eller 3 anonyma enkäter rangordna alla resultat som vi har identifierat.

Vi ska även ha gruppdiskussioner och intervjuer där vi pratar vidare om egna erfarenheter i den könsbekräftande vården. Gruppdiskussioner/intervju genomförs av forskare på Uppsala universitet. Möjlighet finns att delta online.

Kan du tänka dig att delta i de ovan beskrivna enkäterna och/eller en gruppdiskussion/intervju?

Ditt svar är inte bindande. Du kan alltid ändra dig.

◯ Ja
◯ Nej

Vad bra! Klicka då på länken som visas när du har lämnat in dina svar. Du kommer då vidare till en kort enkät där du får ange din e-postadress och ditt intresse av att delta i ytterligare enkäter, en intervju eller en gruppdiskussion.

Din e-postadress kommer INTE kunna kopplas till dina svar i den här enkäten.

Tack så mycket för din hjälp!
