# Supplementary material for: Developing a core outcome set for gender-affirming healthcare in transgender and gender diverse adults in Sweden using the Delphi approach: a study protocol
Source: BMJ Open. 2025 Jul 8;15(7):e098300. doi: 10.1136/bmjopen-2024-098300 (PMC12243596; doi:10.1136/bmjopen-2024-098300)
Supplement: Supplementary file 2 [file bmjopen-2024-098300.reviewer_comments.pdf]

PEER REVIEW HISTORY

BMJ Open publishes all reviews undertaken for accepted manuscripts. Reviewers are asked to complete a checklist review form and are provided with free text boxes to elaborate on their assessment. These free text comments are reproduced below.

ARTICLE DETAILS

Title (Provisional)

Developing a Core Outcome Set for Gender-Affirming Healthcare in Transgender and Gender Diverse Adults in Sweden Using the Delphi Process: a study protocol

Authors

Dahlén, Lisann; Pettersson, Klara; Berglund, Frank; Bodlund, Owe; Dhejne, Cecilia; Elfving, Maria; Frisén, Louise; Halldin-Stenlid, Maria; Holmberg, Jenny; Holmberg, Mats; Högström, Jens; Indremo, Malin; Karvonen, Levi; Kratz, Gunnar; Nygren, Ulrika; Selvaggi, Gennaro; Skalkidou, Alkistis; Summanen, Edward; Södersten, Maria; Tivesten, Åsa; Clark, Kristen D; Papadopoulos, Fotios C.

VERSION 1 - REVIEW

**Reviewer**	**1**
**Name**	**Mullender, Margriet**
**Affiliation Plastic**	**Amsterdam University Medical Centre, Department of**
**Date**	**18-Apr-2025**
**COI**	**None**

This study describes a protocol for the development of a Core Outcome Set (COS) for (all) gender-affirming treatments (GAT). It follows the general methodology for COS development according to the COMET initiative. I applaud the authors for their initiative to standardize outcome measurement in gender-affirming care. This is certainly needed. The multi-stakeholder approach is also thorough, with representation from the transgender and gender diverse community.

However, this study protocol is flawed because the scope of this intended COS development is far too broad and unspecific. It is unclear what the focus is.

The introduction mentions that 'gaps in knowledge' have been identified, without specifying these gaps. The authors refer to articles on psychosocial functioning after hormone therapy, suggesting that these gaps refer to a lack of knowledge about psychosocial functioning in the context of hormone therapy. They also argue that there is a lack of consensus about what outcomes should be measured after GAT, in this case referring to studies of patient-reported

outcome measures after gender-affirming surgery. However, they go on to give the example of a study of risk and protective factors for self-harm in transgender and gender diverse people, mentioning different instruments for measuring depression. It is unclear to me how these three contexts can be combined in one project for COS development.

Furthermore, the purpose of this COS development and the context in which it is to be used is stated as 'GAT provision' and 'clinical research', which is also very unspecific. This complete lack of focus makes this project unfeasible.

Firstly, the authors should clarify what 'gaps' are being addressed by this COS development. Secondly, a realistic scope should be chosen. If the aim of this COS is to specifically address 'gender dysphoria' (i.e., the experienced distress related to gender incongruence) as a health condition, then this should be made explicit. If gender-affirming treatments are of interest, these are too diverse to be captured in one COS. As it is now proposed, it seems, for example, like developing a COS for all urological treatments together. Thirdly, it should also be made clear where and how the COS is to be used.

In line with this, the methods for the umbrella review (Phase 1) are also rather unspecified. How is clinical research on GAT defined? What are the inclusion and exclusion criteria? Recently, two separate systematic reviews have assessed all outcomes reported after masculinizing and feminizing genital gender-affirming surgery, both counting more than 2000 outcomes reported in clinical research. If reviews of genital gender-affirming surgery alone provide this number of outcomes, how will the study team capture the outcomes of all GAT? It should be made clear how the study team expects to deal with the number and diversity of these outcomes.

By the way, it is interesting to note that there is no reference to the protocol for COS development for genital gender-affirming surgery, which falls within the scope of this project.

Lastly, the dates of the study are missing in the manuscript.

VERSION 1 - AUTHOR RESPONSE

Introduction		
	The introduction mentions that 'gaps in knowledge' have been identified, without specifying these gaps. The authors refer to articles on psychosocial functioning after hormone therapy, suggesting that these gaps refer to a lack of knowledge about psychosocial functioning in the context of	We have revised the introduction considerably to more clearly communicate the gaps identified and the relationship with psychosocial outcomes that was mentioned briefly:

Commented [KC1]: Please delete any files that you intend to replace with updated versions and upload the following using the appropriate file designation:
- “Main Document” - This is a clean copy (without tracked or highlighted changes) of your revised article. Please delete your original submission file.
- “Main Document - marked copy” - This is the edited version of your original article, including edits to address the peer review comments. Any changes have been highlighted using a track change function or bold or coloured text.
Please replace any other files that have been updated e.g. Images, forms.

	hormone therapy. They also argue that there is a lack of consensus about what outcomes should be measured after GAT, in this case referring to studies of patient-reported outcome measures after gender-affirming surgery. However, they go on to give the example of a study of risk and protective factors for self-harm in transgender and gender diverse people, mentioning different instruments for measuring depression. It is unclear to me how these three contexts can be combined in one project for COS development. Firstly, the authors should clarify what 'gaps' are being addressed by this COS development.	"A lack of consensus on which essential outcomes should be measured in GAT research and clinical care is one particular concern (9–11). There is also considerable heterogeneity in the measurement of outcomes applied in GAT research and clinical care (9–11) For example, in a systematic review of 78 studies on risk and protective factors for self-harm in TGD people, Bird et al (12) found a diverse range of reported outcomes and measurement instruments, including ten different rating scales for depression. Similarly, a review on the effects of gender-affirming hormonal treatment by The Swedish Agency for Health Technology Assessment and Assessment of Social Services found that different versions of varying rating scales of gender dysphoria were used across and within studies (13). Additionally, psychosocial functioning is often used as an outcome for determining the effectiveness of GAT; however, there is ambiguity on which aspects of this broad construct are relevant and how they should be assessed (6). These

		gaps are interwoven, the lack of consensus on what to measure and how to measure it contributes to a fragmented understanding of psychosocial functioning as well as other outcomes in GAT for TGD individuals. This fragmentation obstructs the advancement of evidence-based treatment guidelines and limits the ability of researchers and clinicians to evaluate the effectiveness of GAT interventions.“ (pgs. 4-5(
	Furthermore, the purpose of this COS development and the context in which it is to be used is stated as 'GAT provision' and 'clinical research', which is also very unspecific. This complete lack of focus makes this project unfeasible. Secondly, a realistic scope should be chosen. If the aim of this COS is to specifically address 'gender dysphoria' (i.e., the experienced distress related to gender incongruence) as a health condition, then this should be made explicit. If gender-affirming treatments are of interest, these are too diverse to be captured in one COS. As it is now proposed, it seems, for example, like developing a COS for all urological treatments together.	We have revised the purpose and gaps to be more explicit as to the purpose of such a broad COS protocol:<br><br>“In accordance with the COMET guidelines, this study aims to develop a foundational COS intended for use across the broad domain of GAT. The purpose of this COS is to establish a base set of outcomes that should be measured and reported in both clinical research and in routine care involving TGD individuals receiving GAT, in contrast to other protocols, such as GenderCOS which sought to identify COS for genital surgeries for TGD

		individuals (16). This COS is not intended to replace more specific and nuanced COS that may be necessary within specialties (e.g., surgery, endocrinology), but rather to complement and unify them by ensuring that key outcomes, particularly those related to psychosocial functioning, mental health, and overall treatment impact, are captured consistently. Subsequently, the scope of this COS is deliberately broad, reflecting the multidisciplinary construction of GAT and the need for a shared framework across specialties. Future COS initiatives may build upon this foundation to develop domain-specific extensions tailored to particular interventions or specialties.“ (pgs. 5-6)
	Thirdly, it should also be made clear where and how the COS is to be used.	We have revised our purpose statement to be clearer on the intended use:<br>“The purpose of this COS is to establish a base set of outcomes that should be measured and reported in both clinical research and in routine care involving TGD individuals receiving GAT, in contrast to other protocols, such as GenderCOS, which sought to identify COS for genital surgeries for TGD individuals (16). This

		COS is not intended to replace more specific and nuanced COS that may be necessary within specialties (e.g., surgery, endocrinology), but rather to complement and unify them by ensuring that key outcomes, particularly those related to psychosocial functioning, mental health, and overall treatment impact, are captured consistently." (pgs. 5-6)
Methods		
	In line with this, the methods for the umbrella review (Phase 1) are also rather unspecified. How is clinical research on GAT defined? What are the inclusion and exclusion criteria? Recently, two separate systematic reviews have assessed all outcomes reported after masculinizing and feminizing genital gender-affirming surgery, both counting more than 2000 outcomes reported in clinical research. If reviews of genital gender-affirming surgery alone provide this number of outcomes, how will the study team capture the outcomes of all GAT? It should be made clear how the study team expects to deal with the number and diversity of these outcomes.	We have added additional wording to clarify the definition of clinical research:<br><br>"An umbrella review using the PubMed database will be conducted to compile a comprehensive list of outcomes typically assessed in clinical research (i.e., studies involving participants in clinical settings) on gender dysphoria." (pg. 8)<br><br>We also added clarification on the inclusion and exclusion criteria:<br><br>"Inclusion criteria are: studies must be a systematic review or umbrella review, involve adult participants, published during the past two years (2023-2024), and published in English, Swedish, or German. Therefore, reviews involving only adolescents or youth (<18 years) were excluded." (pg. 8, p. 3)<br><br>We expanded on the methodology for refining and synthesizing the identified outcomes:<br><br>"Three study personnel will review all outcomes for proposed synthesis, with a fourth individual

		who will resolve any disagreements." (pg. 9, p. 3)
	By the way, it is interesting to note that there is no reference to the protocol for COS development for genital gender-affirming surgery, which falls within the scope of this project.	We have added mention of this COS to the introduction:<br><br>""In accordance with the COMET guidelines, this study aims to develop a foundational COS intended for use across the broad domain of GAT. The purpose of this COS is to establish a base set of outcomes that should be measured and reported in both clinical research and in routine care involving TGD individuals receiving GAT, in contrast to other protocols, such as GenderCOS which sought to identify COS for genital surgeries for TGD individuals (16)." (pg. 5)
Reviewer 1	Lastly, the dates of the study are missing in the manuscript.	We have added the planned start and end dates of the study (see above).
